# Recent Developments in Zn-Based Biodegradable Materials for Biomedical Applications

**DOI:** 10.3390/jfb14010001

**Published:** 2022-12-20

**Authors:** Muzamil Hussain, Sami Ullah, Muhammad Rafi Raza, Naseem Abbas, Ahsan Ali

**Affiliations:** 1 Department of Mechanical Engineering, COMSATS University Islamabad, Sahiwal Campus, Punjab 57000, Pakistan; 2 Department of Chemistry, College of Science, King Khalid University, Abha 61413, Saudi Arabia; 3 Department of Mechanical Engineering, Sejong University, Seoul 05006, Republic of Korea; 4 Department of Mechanical Engineering, Gachon University, Seongnam-si 13120, Republic of Korea

**Keywords:** biodegradable materials, biodegradability, biocompatibility, Zn alloys

## Abstract

Zn-based biodegradable alloys or composites have the potential to be developed to next-generation orthopedic implants as alternatives to conventional implants to avoid revision surgeries and to reduce biocompatibility issues. This review summarizes the current research status on Zn-based biodegradable materials. The biological function of Zn, design criteria for orthopedic implants, and corrosion behavior of biodegradable materials are briefly discussed. The performance of many novel zinc-based biodegradable materials is evaluated in terms of biodegradation, biocompatibility, and mechanical properties. Zn-based materials perform a significant role in bone metabolism and the growth of new cells and show medium degradation without the release of excessive hydrogen. The addition of alloying elements such as Mg, Zr, Mn, Ca, and Li into pure Zn enhances the mechanical properties of Zn alloys. Grain refinement by the application of post-processing techniques is effective for the development of many suitable Zn-based biodegradable materials.

## 1. Introduction

Orthopedic prostheses are used for the repair of bone fractures or the replacement of fractured bones. Several types of prostheses are used according to the severity of bone fractures. Conventionally, metallic devices are used to fix bone fractures, but these devices induce many adverse effects such as bone necrosis, osteoporosis, and delayed bone healing inside the human body [1,2]. Many other problems associated with conventional fixation devices are allergic reactions, the release of ions, corrosion, fatigue failure, the release of hydrogen, stress shielding, and revision surgeries [3,4,5,6,7]. A second surgery is needed to remove the non-degradable device after serving the required function and bone healing. To overcome the problems associated with non-degradable devices, biodegradable prostheses have been introduced. These devices resorb or degrade in the physiological environment over sometime during the healing process [8,9,10,11,12].

Many metal-based and polymer-based materials are well-known options for manufacturing biodegradable prostheses. Among these materials, magnesium-based and Zn-based materials are the most suitable biomaterials for the fabrication of biodegradable devices. Their rapid degradation, as well as the excessive release of degradation products of magnesium-based biomaterials, has limited their use in biomedical applications [13,14,15,16,17]. Biodegradable Zn alloys show medium degradation rates (*DR*) in contrast to magnesium-based biodegradable materials; their biodegradation products are fully biodegradable without releasing excessive hydrogen gas. Compared to magnesium alloys, Zn alloys exhibit a lower corrosion rate because of their lower electrode potential [18,19,20].

To improve the properties of biodegradable materials, researchers are focusing on optimizing the properties of biodegradable materials by making alloys or composites. A huge chunk of the commercial sector is conducting research and investing its resources in developing efficient and effective biodegradable materials for orthopedic implants. From orthopedic to cardiac and from plastic surgery to oncology, the range of applications of these materials is of no limit. Similarly, the awareness, acceptability, and utility of these materials are on a continuous rise. Currently, numerous Zn-based alloys have been utilized by integrating bioactive substances or adjusting material processing methods with an objective on the optimization of their biodegradation and mechanical properties. These materials have the potential to be developed into next-generation orthopedic implants as alternatives to conventional implants, in order to avoid revision surgeries and reduce biocompatibility issues. Several challenges such as controllable biodegradation behavior and comparable mechanical properties need to be overcome for acceptance in the industrial sector.

In recent years, many review articles have been published on Zn-based biodegradable materials. Yuan et al. [21] summarized the surface modification methods for Zn-based biodegradable materials. Li et al. [22] summarized the challenges and opportunities for the development of Zn-based biodegradable materials. Various processing and fabrication methods were discussed. Kabir et al. [23] discussed the biocorrosion and biochemical perspectives of Zn-based biodegradable materials. Shi et al. [24] discussed the effect of the second phase and alloying elements on the mechanical properties of Zn-based biodegradable materials. Huang et al. [25] discussed the effect of alloying elements on the softening phenomenon of Zn-based biodegradable materials. Possible strategies to minimize strain softening were proposed. Yang et al. [26] discussed the effect of Zn and other nutrient elements on the wound-healing process. Chen et al. [27] discussed the challenges in the development of metal-based biodegradable membranes for bone regeneration. In this review, we summarize the current research status on Zn-based biodegradable materials. Many novel Zn-based biodegradable materials developed in recent years are evaluated in terms of their biodegradation, biocompatibility, and mechanical properties. This review will help researchers to make suitable alloy compositions to meet the required clinical demand.

## 2. Biological Functions of Zn

Zn is the second most abundant element in the human body after iron. In the human body, 11% of Zn presents in the liver and skin, 85% of Zn exists in bone and muscles, and the remaining presents in other tissues [28,29,30,31]. Zn plays important role in different biological functions. The presence of Zn plays a significant role in enzymes performing their regulatory or catalytic actions [32,33,34,35]. Zn performs a significant role in bone metabolism and the growth of an organism. Zn supplementation enhances bone formation, meanwhile, increasing bone strength by stimulating osteoblast and differentiation of osteoclast [36,37,38]. Zn deficiency is associated with the weakness and health of bones.

The addition of Zn into biodegradable materials can enhance osteoblast differentiation by promoting bone marrow genes such as osteopontin, osteocalcin, collagen, and alkaline phosphatase [39]. Zn acts as a strong inhibitor of osteoclastic bone resorption as compared to other metals. Furthermore, Zn performs a significant role in protection against cardiomyopathy and heart disease. Zn supplementation can improve cardiac function and prevents damage in case of infarction and ischemia. Zn is important in maintaining the integrity of normal endothelial cells. In addition, it can also stimulate endothelial cell proliferation by enhancing basic growth factor-dependent endogenous fibroblast proliferation. Zn is also involved in the integrity and development of the immune system. Zn has a significant impact on the activity of certain important immune mediators composed of cytokines, thymic peptides, and enzymes [40]. For its part, Zn is essential for the intracellular regulation of lymphocyte apoptosis. Zn is involved in neurotransmission, neuronal growth, synaptogenesis, and neurogenesis. It is selectively stored in the presynaptic vesicles of specific neurons and released as a neuromodulator.

**Figure 1:**
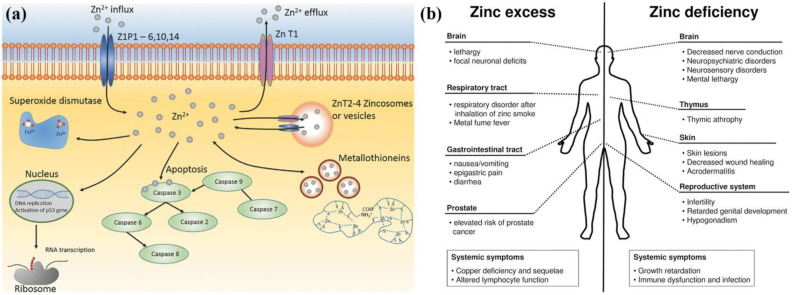
Biological functions and roles of Zn in the human body (**a**) Biological functions of Zn (**b**) Effect of Zn excess and deficiency in the human body [21].

Although Zn is essential for many physiological functions, excessive Zn exposure or intake can have adverse effects on various organs in addition to insufficient Zn intake. Zn deficiency can lead to various pathological symptoms, including growth disorders, birth defects, and hypotension, among others. Many diseases are also associated with Zn deficiency, such as gastrointestinal diseases, kidney diseases, sickle cell disease, etc. On the other hand, an excess of Zn can also have detrimental consequences. Zn^2+^ is capable of inhibiting electron transport in uncoupled mitochondria. It is teratogenic or lethal for embryogenesis in case of excessive intake of Zn. Zn^2+^ is now reported to have a biphasic effect on cell viability, adhesion, and proliferation. A high concentration of Zn^2+^ would lead to a suppressive effect on cytocompatibility. Figure 1 presents the biological functions and roles of Zn in the human body.

## 3. Design Criteria for Orthopedic Devices

The most important characteristics of the biodegradable devices are their biodegradability, biocompatibility, mechanical properties, corrosion behavior, and antibacterial activity. The biodegradable device should be hypoallergenic, non-inflammatory, and non-toxic with no harmful retention or release of particulates [41,42,43,44]. The biodegradable device must be capable to promote the growth of new cells and bone generation. The mechanical properties, such as ultimate tensile strength ($\sigma_{UTS}$) > 300 MPa, tensile yield strength ($\sigma_{TYS\;})\;$> 230 MPa, and elongation (δ) > 15–18%, are required, and the elastic modulus (*E*) should be similar to bone (10–20 GPa) [45,46]. The service time of a device must be equal to 1–2 years for performing the particular function till full absorption of a device. The integrity of a device must be equal to 3 to 6 months for screws, pins, and staples [21]. Another important concern is the corrosion behavior of a biodegradable device. In vitro corrosion test experiments should show a degradation/penetration rate (DR) < 0.5 mm/year and hydrogen evolution should be less than 10 µL/cm^2^-day.

## 4. In Vivo Corrosion

The corrosion occurs on the implantation of biodegradable materials in a physiological environment through the degradation process, which may result in health issues due to the formation of H_2_ gas and the release of metal ions [47]. Therefore, shifting of the pH region in the surrounding corroding surface is an important concern for orthopedic applications [48,49,50,51]. Generally, in the corrosion mechanism of metal-based biodegradable devices, the metals are oxidized into cations and H_2_, hydroxides, and oxides are produced by electrochemical reactions [52,53,54]. Finally, the metal oxide layer is formed on the surface of biodegradable metals, which acts as a kinetic barrier or passive layer and prevents the further electrochemical reaction or release of ions across the substrate’s surface [53,55]. However, this metal oxide layer can be dissolved in the electrolyte, and the pitting corrosion process starts after it [56,57]. Pitting is localized corrosion and occurs with the breakdown of the passive film. This form of corrosion harms biodegradable material, as it is not easy to observe the pits on the biodegradable material surface in an aggressive environment due to the presence of corrosion products. After the initiation of pitting corrosion, biodegradable materials corrode rapidly and the load-carrying capability of the implant is reduced. Additionally, the increase in localized stress due to pitting has the potential to produce cracks, and the implant may fail due to stress corrosion and fatigue cracking within the pits. So, the rate of evolved H_2_ should be minimum to control degradability.

In vitro electrochemical and immersion tests are used to evaluate the corrosion behavior of biodegradable implants. In these physiological environments, biodegradable metals are susceptible to corrode due to their electrochemical potential. Corrosion current density ($I_{corr}$) and corrosion potential ($E_{corr}$) are measured in electrochemical tests. The corrosion in vitro and in vivo environment is influenced by many factors such as types of released ions, pH concentration, biological response of surrounding tissues, and protein absorption on the implant surface. The condition of corroding implant material can be assessed by monitoring the amount of released ions. The pH is monitored in immersion tests to assess the corrosion rate (CR) of biodegradable material. The lower pH value indicates a lower corrosion rate and an increasing pH value is unfavorable for cell adhesion. Fast corrosion may cause structural failure, unwanted degradation, alkaline pH shift, and hydrogen evolution in the surrounding corroded sites [54].

## 5. Zn-Based Biomaterials

Zn-based biodegradable materials are receiving attention for orthopedic applications due to their good combination of biocompatibility and degradability. The present Zn-based alloys are not sufficiently biocompatible, nor necessarily wear-resistant and mechanically strong [58]. Pure Zn materials show poor mechanical characteristics, and they cannot be used for most orthopedic applications. In addition, the relatively low creep resistance, low fatigue strength, high susceptibility, and low-temperature recrystallization of Zn has limited its use for the development of implant materials. In recent years, many alloys or composites of Zn-based biodegradable materials have been established with improved biocompatibility, bio-corrosion, and mechanical properties [59,60,61,62,63]. Many essential trace elements for the human body have been used for making Zn-based biodegradable alloys, and many types of reinforcement materials have been used for making Zn composites [64,65,66,67]. Among these reinforcements, calcium phosphate-based reinforcements are the most widely used [41]. Many types of fabrication methods such as casting, powder metallurgy, transient directional solidification, additive manufacturing, spark plasma sintering, or other advance processing techniques are used for making alloys or composites of Zn [68,69,70,71,72,73,74]. Among the different fabrication methods, casting is the most common method for the mass production of Zn-based alloys.

**Figure 2:**
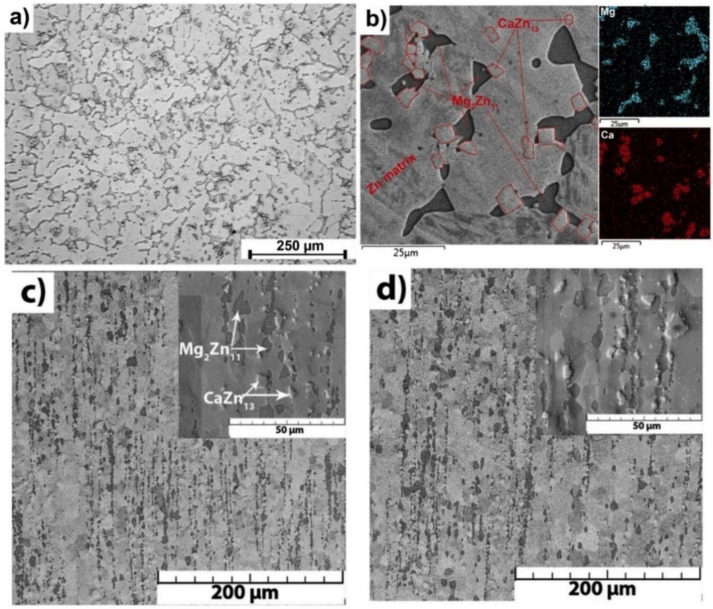
Microstructures of Zn–0.8Mg–0.2Ca alloys (**a**) Microstructure of as-cast (cast and annealed) alloy; (**b**) a detailed SEM view with corresponding X-ray elemental maps of Mg and Ca; (**c**) SEM image of extruded material at 300 °C and an extrusion ratio of 11:1; (**d**) SEM image of extruded material at 300 °C and an extrusion ratio of 25:1. Reprinted with modification and permission from [75].

Zn-based alloy compositions are multiphase systems, and their mechanical, degradation, and corrosion behaviors are strongly dependent on the microstructural parameters and the distribution of the secondary phase in the alloy matrix. Refined microstructures and uniform distribution of the second phase throughout the alloy composition are expected to result in improved properties of biodegradable Zn alloys. The microstructures and resultant mechanical properties of Zn-based materials can be tailored by the application of various conventional metal-forming processing techniques such as hot extrusion, rolling, selective laser method (SLM), spark plasma sintering (SPS), drawing, and forging, and severe plastic deformation techniques such as equal channel angular pressing (ECAP), high-pressure torsion, twist extrusion, friction-stir processing, cylinder-covered compression, and multi-directional forging. The grain refinement achieved in post-processing techniques improves their corrosion resistance and mechanical properties. It is difficult to study the influence of post-processing techniques on the mechanical characteristics of Zn alloys due to the small sizes of processed Zn-based materials or the softening of Zn-based materials at high strains as a result of dynamic recrystallization. Capek et al. [75] studied the influence of extrusion parameters such as the extrusion ratio and temperature on the microstructure and mechanical properties of Zn–0.8Mg–0.2Ca alloys. The microstructures of the as-cast and extruded Zn–0.8Mg–0.2Ca alloys are shown in Figure 2. The Zn-based metallic matrix contains coarse grains (grain size varying between 100 and 40 µm) and particles of Mg_2_Zn_11_ (dark in SEM image with a grain size of 8.3 µm) and CaZn_13_ (encircled by red lines in the SEM image with a grain size of 5 µm) intermetallic phases. The intermetallic particles exist mainly at the Zn grain boundaries and in the internal Zn grain. The presence of α-Zn dendrites and intermetallic phases (Mg_2_Zn_11_ and CaZn_13_) was confirmed by XRD and EDX analyses. The XRD results shown in Figure 2b confirmed that the as-cast alloy contains 85 wt.% of Zn, 10 wt.% of Mg_2_Zn_11,_ and 5 wt.% of CaZn_13_. The microstructures of extruded materials as shown in Figure 2c,d indicate the improvement in microstructure due to the recrystallization effect. The results clearly show that the sizes of both the Zn matrix and intermetallic particles were significantly influenced by the extrusion conditions.

**Figure 3:**
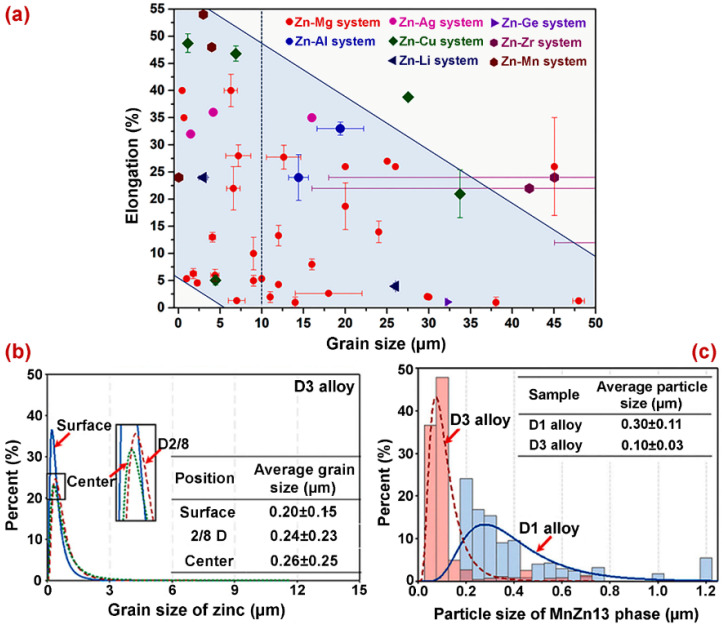
(**a**) The relationship between elongation and grain size for biodegradable Zn alloy, and the grain size distribution of (**b**) Grain sizes of Zn alloys and (**c**) Grain sizes of ZnMn phase. Reprinted with modification and permission from [76].

The refinement of microstructure leads to an enhancement of mechanical properties. The influence of grain size on the elongation of Zn-based biodegradable materials is presented in Figure 3a. Guo et al. [76] performed experiments to improve the microstructure to achieve improved mechanical and degradation properties. The grain size was refined by multi-pass drawing. First, the as-cast alloy samples were preheated and extruded. Then, the extruded alloy samples were cooled, and a deformation method multi-pass drawing was performed. The results suggest that plastic deformation affected the grain size effectively. A significant reduction in grain size was achieved by increasing the amount of deformation. The results suggest that the multi-pass drawing had the potential to alter the MnZn phase size, location, and distribution. The grain sizes of both the Zn and MnZn phases are shown in Figure 3b,c.

**Figure 4:**
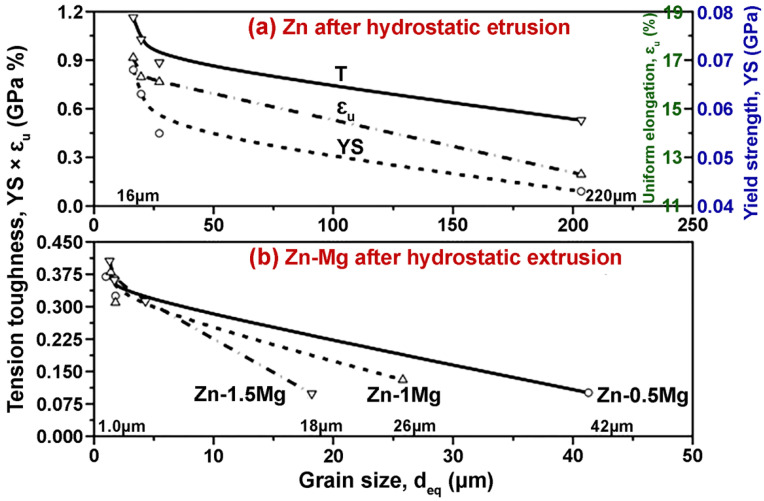
Variation in toughness with grain size distribution for (**a**) Zn and (**b**) Zn–Mg alloys. Reprinted with modification and permission from [80].

Among many Zn-based materials, the Zn–Mg alloys are expected to become potential candidates for orthopedic applications with improved biocompatibility and mechanical properties. The addition of Mg to Zn matrices resulted in the formation of hypoeutectic microstructures. These microstructures are comprised of α-Zn dendrites and a eutectic mixture of α-Zn and Mg_2_Zn_11_ phases [71,77,78,79]. The presence of intermetallic particles (Mg_2_Zn_11_) due to the addition of Mg in Zn significantly enhanced the mechanical properties of Zn matrices. To improve the microstructure and to reduce the grain size of Zn–Mg binary alloy compositions, Pachla et al. [80] performed the hydrostatic extrusion on hot extruded samples of Zn–Mg alloys. The alloy compositions were prepared by gravity casting under an argon atmosphere. The as-cast samples were conventionally extruded at 250 °C. Then, the samples were hydrostatically extruded to reduce the grain size and to compose both alloy phases. The highest degree of refinement was achieved by deformation and the synergistic effect of cumulative hydrostatic extrusion. It was suggested that three to four passes of hydrostatic extrusion are effective for minimizing the maximum temperature of the plastic deformation process. The uniform distribution of alloy phases plays a more important role in the enhancement of mechanical properties. The variation in toughness, with varying grain sizes for Zn-based alloys, is presented in Figure 4.

Guan et al. [81] prepared Zn–2Fe–WC nanocomposites by adding 8 v.% of WC nanoparticles in ZN-2Fe alloy systems using stir casting and ultrasound processing. The deformation process of hot rolling was carried out to improve the mechanical properties. The ultimate tensile stress was increased from 121.1 to 155.8 MPa and elongation was increased from 8.6 to 15.3% as the result of hot rolling. The enhanced mechanical properties of hot-rolled specimens are attributed to the improved porosity and dispersion of nanoparticles. Moreover, immersion and electrochemical tests were carried out to study biocompatibility and the corrosion of composites. The study reveals that WC particles are non-reactive and inert in the physiological environment with no leached W ions. The cytotoxicity results showed that WC nanoparticles exhibit no toxicity to cell lines.

Many post-processing deformation methods have been used to improve the microstructure of Zn-based biodegradable materials. Among these, hot extrusion, hot rolling, and ECAP are most effective to improve the microstructure and reducing the grain size. Therefore, few comparative studies on these deformation methods have been performed to find the optimized method. Huang et al. [82] studied the influence of extrusion, rolling, and ECAP on the microstructural and mechanical performance of Zn–Mg alloys. The maximum improvement in ductility and strength was achieved using ECAP. The influence of multiple passes was also studied. The improved properties were achieved for eight passes as compared to four passes. There are only limited studies on the comparison of different deformation methods. It is difficult to select the optimized deformation method based on the available comparative studies. Among different deformation methods, hot extrusion is the most widely used method for improving the microstructure of Zn-based alloys.

**Figure 5:**
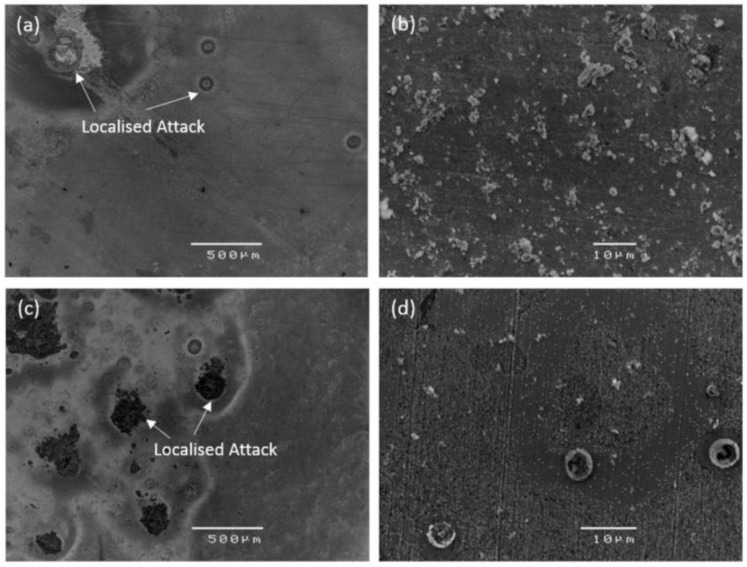
SEM images of Zn alloys after the immersion tests; (**a**) 500 µm resolution Zn image; (**b**) 10 µm resolution Zn image; (**c**) 500 µm resolution Zn–5Al–4Mg alloy image; (**d**) 10 µm resolution Zn–5Al–4Mg alloy image. Reprinted with changes and permission from [83].

The mechanical stability of orthopedic prostheses is an important concern that is highly dependent on corrosion behavior. Kannan et al. [83] compared the degradation characteristics and biocompatibility of Zn and Zn–5Al–4Mg alloys. Using in vitro corrosion, the Zn alloy samples were immersed in SBF solution for a period of seven days. The SEM images of immersed samples are shown in Figure 5. The SEM images verify the limited corrosion strike on both Zn alloys. The degradation behavior with the function of immersion time was analyzed. The degradation rate of Zn was recorded as being less when compared to Zn–5Al–4Mg alloy.

**Figure 6:**
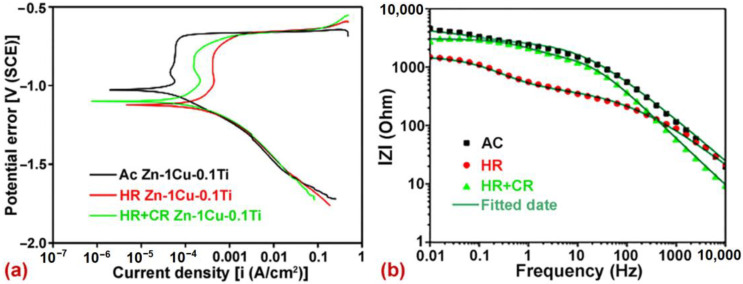
Immersion test results of Zn alloys: (**a**) polarization curves and (**b**) bode impedance modulus curves. Reprinted with changes and permission from [58].

In vitro and in vivo studies are performed to analyze the corrosion and degradation performance of Zn-based biodegradable materials. Lin et al. [58] developed different compositions of Zn–1Cu–0.1Ti alloys by casting. The rolled alloy specimens were compared with as-cast specimens, and various parameters were investigated (including mechanical properties, corrosion resistance, biocompatibility, and antibacterial ability). Hot-rolled specimens exhibit improved mechanical performance. The corrosion behavior was assessed from the polarization curves of alloys, which are displayed in Figure 6a. The maximum corrosion was recorded for hot-rolled specimens in terms of both the corrosion current and corrosion density. The minimum passive layer formation on the surface of Zn alloys was associated with the decreased dissolution rate. Figure 6b illustrates the impedance for all Zn alloys. The larger values of impedance indicate an improvement in corrosion resistance.

**Figure 7:**
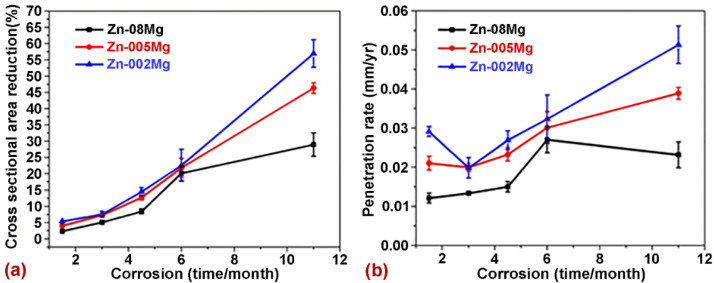
Degradation behavior: (**a**) cross-sectional area reduction and (**b**) penetration rate. Reprinted with modification and permission from [84].

Jin et al. [84] developed the different Zn–Mg alloy compositions including the Zn–0.08Mg, Zn–0.005Mg, and 0.002Mg. The as-cast alloys were further extruded and drawn to improve the microstructure. In vivo studies were performed using Sprague-Dawley rats. The samples were placed within the arterial extracellular matrix for a period of 1.5, 3, 4.5, 6, and 11 months. The cross-sectional area reduction and penetration rate were measured to access degradation behavior. The degradation behavior in terms of cross-sectional area and penetration rate is shown in Figure 7. The degradation rate values evaluated from the penetration rate were higher for all compositions but close to the benchmark value (0.02 mm/y).

**Figure 8:**
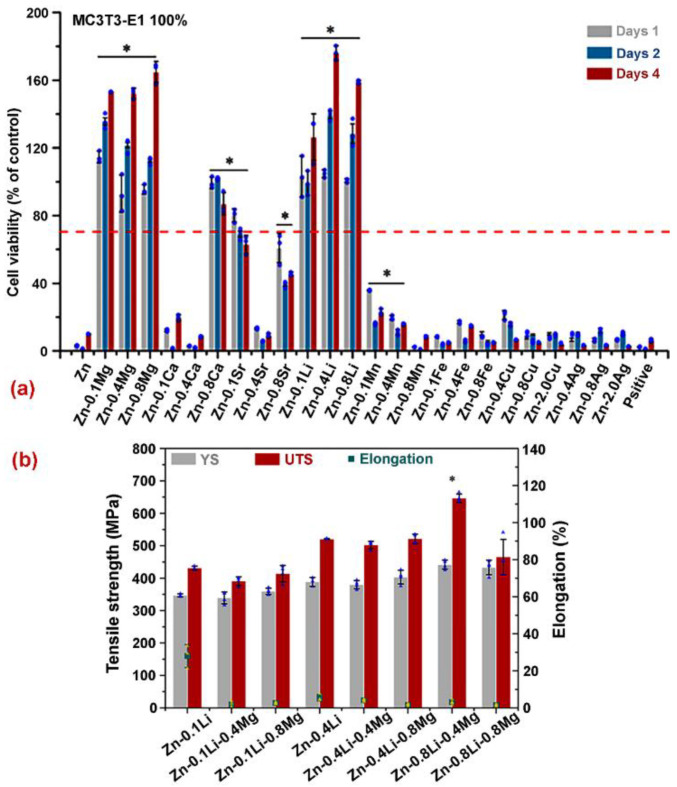
(**a**) Cell viability of binary Zn-based alloys using MC3T3-E1 cells; (**b**) Mechanical properties of ternary Zn alloys. * *p* value < 0.05 by one-way ANOVA with Tukey’s post hoc test. Reprinted with changes and permission from [85].

Yang et al. [85] fabricated the twenty-four binary Zn alloy compositions of different eight elements such as Cu, Ca, Mn, Sr, Ag, Fe, Mg, and Li. The extrusion was performed for improving the Zn microstructure. First, the superior compositions of alloys were screened in mechanical and in vitro tests. Then, the selected samples were tested in vivo through application into the rat femur. Zn–Li and Zn–Mn alloys exhibited the highest ductility and tensile strength. Zn–Mn alloys exhibited improved corrosion properties as compared to other compositions. The growth of new tissues was noticed in cell viability tests. The results of the study are shown in Figure 8a. Yang et al. [85] also developed nine ternary Zn alloy compositions based on the optimized binary composition of Zn–Li. The different weight fractions of Mg and Mn were added to optimize the properties of the Zn–Li binary alloy. The maximum enhancement in mechanical properties was achieved for two ternary alloy compositions including Zn–0.8Li-0.4Mg and Zn–0.8Li-0.8Mn. The mechanical properties of ternary Zn alloys are shown in Figure 8b.

The tribological nature of Zn-based biomaterials is not much reported in the literature. Currently, Lin et al. [58] performed tribological studies on a Zn–1Cu–0.1Ti alloy. The friction and wear behavior of as-cast, hot-rolled, and cold-rolled Zn–1Cu–0.1Ti alloys showed that the hot-rolled Zn–1Cu–0.1Ti alloy exhibited the best tribological performance. A few other research groups also reported the results of tribological studies for Zn-based biomaterials, but the studies of wear on Zn-based biomaterials are limited and there is a need to perform the wear studies before the clinical trials. The biodegradation, mechanical, biocompatible, and tribological results on Zn-based biomaterials are presented in Table 1. A broad range of alloying elements, such as Mg, Mn, Fe, Ca, Cu, Li, Ag, Al, Ge, Sr, Zr, and Ti are used for making Zn alloys. The results in Table 1 clearly show that pure Zn exhibits fewer mechanical properties and does not meet the required design criteria. Zn–Mg alloys exhibit good mechanical properties and meet the required design criteria for orthopedic implants. Zn–Cu also exhibits good mechanical properties but the presence of Cu makes these alloys unsuitable due to the impropriate biological properties of Cu. Among different alloying elements, the addition of Li into pure Zn enhanced the mechanical properties of Zn-based alloys. The results in Table 1 show that the ternary alloy systems such as Zn–xLi–yMn (x, y = 0.1–0.8 wt.%) are the best candidates for next-generation orthopedic devices.

## 6. Conclusions

There is an increasing demand for innovative clinical orthopedic implants for aging-related bone diseases. Zn-based materials can meet the required design criteria by adding the alloying elements and refining the microstructure by applying post-processing deformation methods. Zn-based biodegradable materials may be important orthopedic implants to treat challenging bone diseases, attributed to their desired mechanical and degradation properties. This review summarizes the biological function of Zn, the design criteria for orthopedic implant materials, and the performance of Zn-based biodegradable alloys. The following points were concluded:

- Zn exists in bones and muscles in the human body and performs a significant role in bone metabolism and the growth of an organism. Zn-based biodegradable materials can enhance osteoblast differentiation by promoting bone marrow genes.
- To meet the design criteria of a biodegradable device, mechanical properties such as ultimate tensile strength ($\sigma_{UTS}$) > 300 MPa, tensile yield strength ($\sigma_{TYS\;})\;$> 230 MPa, and elongation (δ) > 15–18% are required, and the elastic modulus (*E*) should be similar to bone (10–20 GPa). The service time of a device must be equal to 1–2 years for performing the particular function until the full absorption of the device. In vitro corrosion test degradation/penetration rate should be (*DR*) < 0.5 mm/year and hydrogen evolution should be less than 10 µL/cm^2^-day.
- Using in vitro corrosion, Zn-based biodegradable materials show medium degradation rates and are oxidized into hydroxides and oxides without releasing excessive hydrogen gas.
- Zn-based alloys are multiphase systems, and their mechanical and degradation properties are strongly dependent on the grain sizes and the distribution of the secondary phase in the alloy matrix. Refined microstructures and uniform distribution of the second phase throughout the alloy composition are expected to result in improved properties of biodegradable Zn alloys.
- Many post-processing methods have been used to improve the microstructure of Zn-based biodegradable materials. Among these, hot extrusion, hot rolling, and ECAP are the most effective to improve the microstructure and reducing the grain size. The grain refinement achieved in post-processing techniques improves their corrosion resistance and mechanical properties.
- Zn–Mg alloys exhibit good mechanical properties and meet the required design criteria for orthopedic implants. The addition of Li into pure Zn enhances the mechanical properties of Zn-based alloys. The ternary alloy systems such as Zn–xLi–yMn (x, y = 0.1–0.8 wt.%) are the best candidates for next-generation orthopedic devices.
- There is a need to test the most suitable Zn-based biodegradable materials in all aspects before the clinical trial. The biocompatibility studies in vivo and tribological studies are limited to Zn-based biodegradable materials.
- Many suitable combinations of Zn-based biodegradable materials are listed based on the results of previous studies. Still, these materials are not used in orthopedics. There is a need to study the factors which make their use limited.

## Figures and Tables

**Table 1:** Biodegradation, mechanical, biocompatible, and tribological results of several studies on Zn-based biomaterials.

Material	Processing Method (Grain Size)	Corrosion Test Results	Mechanical Test Results	Tribological Results/Biocompatibility	Ref.
Zn	Hot Extrusion (14)	$E_{corr}—$−0.098 V $I_{corr}—$8.9 µA/cm^2^ DR—0.133 mm/y (14)	$\sigma_{TYS}—$55 MPa $\sigma_{UTS}$—97 MPa δ—7.7%	Nr	[86]
Zn	Hot Extrusion (151 µm)	$E_{corr}$—−0.98 V $I_{corr}—$8.98 µA/cm^2^ DR—0.134 mm/y (14)	$\sigma_{TYS}—$51 MPa $\sigma_{UTS}$—111 MPa δ—60% H—34 HV	Nr	[87]
Zn	Hot rolling	$E_{corr}$—−1.077 V $I_{corr}—$20.9 µA/cm^2^ DR—0.306 mm/y (14)	$\sigma_{TYS}—$35 MPa $\sigma_{UTS}$—49 MPa δ—6% H—40 HV	Nr	[88]
Zn	Selective laser method (104 µm)	$E_{corr}$—−0.87 V $I_{corr}—$9.24 µA/cm^2^ DR—0.18 mm/y (28)	$\sigma_{TYS}—$43 MPa $\sigma_{UTS}$—61 MPa E—12 GPa δ—1.7% H—50 HV	Nr	[89]
Zn-25Mg	Powder Metallurgy	DR—0.374 mm/y $E_{corr}—$−1.323 V $I_{corr}—$12.2 µA/cm^2^ (2)	E—86 GPa δ—5.2% $\sigma_{CYS}$—403 MPa H—86 HV	Nr	[74]
Zn-1Mg	Hot Extrusion (4.4 µm)	$E_{corr}—$−1.07 V $I_{corr}—$11.8 µA/cm^2^ DR—0.177 mm/y (14)	$\sigma_{TYS}—$180 MPa $\sigma_{UTS}$—340 MPa δ—6% H—75 HV	Nr	[87]
Zn-0.8Mg	Hot Extrusion (20 µm)	DR—0.071 mm/y (1)	$\sigma_{TYS}—$203 MPa $\sigma_{UTS}$—301 MPa δ—13% $\sigma_{CYS}$—186 GPa H—83 HV	Nr	[86]
Zn-0.5Mg	Hydrostatic Extrusion	Nr	$\sigma_{UTS}$—515 MPa $\sigma_{TYS}—$375 MPa δ—10.5% H—107 HV $\sigma_{UCS}$—473 MPa	Nr	[80]
Zn-1.6Mg	ECAP	$I_{corr}—$6.91 µA/cm^2^ DR—9.31 mm/y	$\sigma_{UTS}$—474 MPa δ—7%	Nr	[82]
Zn-3Mg	Selective laser method	DR—0.1 mm/y (28)	$\sigma_{UTS}$—222 MPa $\sigma_{TYS}—$152 MPa	Cytotoxic at 100% concentration of extract	[89]
Zn-1Mg	Hydrostatic extrusion	Nr	$\sigma_{UTS}$—435 MPa $\sigma_{TYS}—$335 MPa	Nr	[90]
Zn-0.008Mg	Extrusion + Drawing	Nr	$\sigma_{UTS}$—339 MPa $\sigma_{TYS}—$221 MPa	Nr	[84]
Zn-0.005Mg	Indirect Extrusion	DR—0.15 mm/y (14)	$\sigma_{UTS}$—225 MPa $\sigma_{TYS}—$160 MPa δ—26%	Cytotoxic at 100% concentration of extract	[91]
Zn-0.002Mg	Extrusion + Drawing	Nr	$\sigma_{UTS}$—455 MPa $\sigma_{TYS}—$388 MPa	Nr	[92]
Zn-0.05Mg	Hot extrusion (20 µm)	$E_{corr}$—−0.938 V $I_{corr}—$49.1 µA/cm^2^ DR—0.653 mm/y (14)	$\sigma_{TYS}—$160 MPa $\sigma_{UTS}$—225 MPa δ—26%	Nr	[91]
Zn-3Mg	2 Pass ECAP (1.8 µm)	$E_{corr}$—−0.893 V $I_{corr}—$3.2 µA/cm^2^ DR—0.28 mm/y (14)	$\sigma_{TYS}—$205 MPa $\sigma_{UTS}$—220 MPa δ—6.3% E—210 GPa H—186 HV	Nr	[93]
Zn-1.2Mg	Hot Extrusion	$I_{corr}$—−1.18 V $I_{corr}—$7.68 µA/cm^2^ DR—0.12 mm/y (90)	$\sigma_{TYS}—$220 MPa $\sigma_{UTS}$—363 MPa δ—21% H—96 HV	Nr	[94]
Zn-0.5Mn	Multi-pass drawing	DR—0.5 mm/y	$\sigma_{UTS}$—127.6 MPa δ—245%	Good but decrease in biocompatibility	[76]
Zn-4Mn	Nr	$I_{corr}—$48 µA/cm^2^ DR—0.72 mm/y	$\sigma_{UTS}$—298 MPa δ—14.9%	Nr	[72]
Zn-0.1Mn	Extrusion	Change in volume—95% DR—0.014 mm/y	$\sigma_{UTS}$—175 MPa $\sigma_{TYS}—$125 MPa δ—40% $\sigma_{UCS}$—390 MPa $\sigma_{CYS}$—110 MPa H—55 HV	Nr	[85]
Zn-0.8Mn	Hot Extrusion	$E_{corr}$—−0.976 V $I_{corr}—$7.43 µA/cm^2^ DR—0.111 mm/y (30)	$\sigma_{TYS}—$162 MPa $\sigma_{UTS}$—215 MPa δ—44% $\sigma_{CYS}$—136 MPa H—58 HV	Nr	[95]
Zn-0.3Fe	Casting (7.5 µm)	$E_{corr}—$−1.01 V $I_{corr}—$7.31 µA/cm^2^ DR—0.111 mm/y	$\sigma_{TYS}—$70.5 MPa $\sigma_{UTS}$—76.4 MPa δ—1.18% $\sigma_{CYS}$—117 MPa	Nr	[96]
Zn-1.3Fe	Casting	$E_{corr}$—−1.02 V $I_{corr}—$0.67 µA/cm^2^ DR—0.01 mm/y (20)	$\sigma_{TYS}—$80 MPa $\sigma_{UTS}$—134 MPa δ—1.8% H—56 HV	Nr	[97]
Zn-4Cu	Hot Extrusion (2.3 µm)	----	$\sigma_{TYS}—$227 MPa $\sigma_{UTS}$—271 MPa δ—51%	Nr	[98]
Zn-4Cu	Hot Rolling (40 µm)	DR—0.13 mm/y (40)	$\sigma_{TYS}—$327 MPa $\sigma_{UTS}$—393 MPa δ—44.6% $\sigma_{CYS}$—300 MPa H—94 HV	Nr	[99]
Zn-4Cu	Extrusion	DR—0.0255 mm/y (14)	$\sigma_{UTS}$—270 MPa $\sigma_{TYS}—$227 MPa $\sigma_{UTS}$—50.6%	Nr	[98]
Zn-0.1Li	Extrusion + Drawing	Nr	$\sigma_{UTS}$—274 MPa δ—17%	Nr	[100]
Zn-6Li	Hot rolling	$I_{corr}—$3.8 µA/cm^2^ DR—0.05 mm/y	$\sigma_{UTS}$—569 MPa $\sigma_{TYS}—$478 MPa δ—2.4%	Nr	[101]
Zn-0.4Li	Extrusion	DR—0.002 mm/y	$\sigma_{UTS}$—520.36 MPa $\sigma_{TYS}—$390 MPa δ—6% $\sigma_{UCS}$—795 MPa $\sigma_{CYS}$—415 MPa H—165 HV	Cell viability—120% (4)	[85]
Zn-0.4Li	Hot Rolling (10 µm)	$E_{corr}$—−1.21 V $I_{corr}—$3.80 µA/cm^2^ DR—0.05 mm/y (14)	$\sigma_{TYS}—$425 MPa $\sigma_{UTS}$—440 MPa δ—14% H—137 HV	Nr	[101]
Zn-0.4Li	Hot Extrusion	$E_{corr}—$−1.03 V $I_{corr}—$11.26 µA/cm^2^ DR—0.019 mm/y (30)	$\sigma_{TYS}—$387 MPa $\sigma_{UTS}$—520 MPa δ—5% $\sigma_{CYS}$—434 MPa H—164 HV	Nr	[85]
Zn-6Ag	Selective laser method (25 µm)	$E_{corr}—$−0.94 V $I_{corr}—$9.56 µA/cm^2^ DR—0.15 mm/y (21)	$\sigma_{CYS}$—267 MPa H—78 HV	Nr	[73]
Zn-2Ag	Hot extrusion	$E_{corr}—$−1.06 V $I_{corr}—$17.27 µA/cm^2^ DR—0.018 mm/y (30)	$\sigma_{TYS}—$186 MPa $\sigma_{UTS}$—231 MPa δ—36.7% $\sigma_{CYS}$—145 MPa H—55 HV	Nr	[85]
Zn-1Al	Hot Extrusion (14.4 µm)	$E_{corr}—$−0.98 V $I_{corr}—$9.70 µA/cm^2^ DR —0.145 mm/y (14)	$\sigma_{TYS}—$113 MPa $\sigma_{UTS}$—223 MPa δ—24% H—73 HV	Nr	[87]
Zn-2Al	Laser powder bed fusion (5.53 µm)	$E_{corr}—$−1.059 V $I_{corr}—$8.04 µA/cm^2^ DR—0.142 mm/y (14)	$\sigma_{TYS}—$142 MPa $\sigma_{UTS}$—192 MPa E—65 GPa δ—12%	Nr	[102]
Zn-5Al	Hot rolling		$\sigma_{UTS}$—308 MPa δ—16%	Nr	[103]
Zn-5Ge	Hot Extrusion	$E_{corr}—$−0.1063 V $I_{corr}—$10.7 µA/cm^2^ DR—0.157 mm/y (14)	$\sigma_{TYS}—$175 MPa $\sigma_{UTS}$—237 MPa δ—22% H—60 HV		[104]
Zn-3Cu-1Mg	Extrusion	$I_{corr}—$12.4 µA/cm^2^ DR—0.18 mm/y	$\sigma_{UTS}$—441 MPa $\sigma_{TYS}—$427 MPa δ—0.9%	Nr	[105]
Zn-0.5Al-0.5Mg	Nr	$E_{corr}$—−1.018 V $I_{corr}—$9.5 µA/cm^2^ DR—0.12 mm/y (30)	$\sigma_{UTS}$—102 MPa δ—2.1% H—94 HV	Nr	[70]
Zn-3Cu-1Fe	Extrusion	$I_{corr}—$8.8 µA/cm^2^ DR —0.13 mm/y	$\sigma_{UTS}$—272 MPa $\sigma_{TYS}—$221 MPa δ—19.6%	Nr	[106]
Zn-0.8Li-0.8Mg	Hot Extrusion	Nr	$\sigma_{TYS}—$438 MPa $\sigma_{UTS}$—646 MPa δ—3.68%	Nr	[85]
Zn-0.8Li-0.8Mn	Hot Extrusion	Nr	$\sigma_{TYS}—$357 MPa $\sigma_{UTS}$—513 MPa δ—103.5%	Nr	[85]
Zn-1.5Mg-0.5Ca	Hot Extrusion (10–20 µm)	$E_{corr}—$−1.18 V $I_{corr}—$2.08 µA/cm^2^ DR—0.024 mm/y	$\sigma_{TYS}—$160 MPa $\sigma_{UTS}$—442 MPa δ—4.9% H—111 HV	Nr	[107]
Zn-0.02Mg-0.02Cu	Hot Extrusion (13 µm)	DR—0.079 mm/y (15)	$\sigma_{TYS}—$216 MPa $\sigma_{UTS}$—262 MPa δ—28% H—74 HV	Nr	[108]
Zn-1Mg-0.1Sr	Hot Rolling	$E_{corr}—$−1.19 V $I_{corr}—$10.2 µA/cm^2^ DR—0.15 mm/y	$\sigma_{TYS}—$197 MPa $\sigma_{UTS}$—300 MPa δ—23% H—104 HV	Nr	[109]
Zn-1Mg-0.1Mn	Hot Rolling	$E_{corr}$—−1.21 V $I_{corr}—$16.7 µA/cm^2^ DR—0.25 mm/y	$\sigma_{TYS}—$195 MPa $\sigma_{UTS}$—299 MPa δ—26.1% H—108 HV	Nr	[110]
Zn-1Mg-0.1 Zr	Hot Extrusion	$E_{corr}—$−1.23 V $I_{corr}—$5.44 µA/cm^2^ DR—0.23 mm/y (90)	$\sigma_{TYS}—$248 MPa $\sigma_{UTS}$—314 MPa δ—2.5% $\sigma_{CYS}$—300 MPa H—94 HV	Nr	[111]
Zn-2Cu-0.1Ti	Casting	$E_{corr}—$−1.164 V $I_{corr}—$2.56 µA/cm^2^ DR—0.022 mm/y (30)	$\sigma_{TYS}—$132 MPa $\sigma_{UTS}$—177 MPa δ—2.5%	Nr	[112]
Zn-1Cu-0.1Ti	Hot rolling + Cold rolling	DR—0.991 mm/y $E_{corr}—$−1.100 V $I_{corr}—$67.7 µA/cm^2^	$\sigma_{TYS}—$204.2 MPa $\sigma_{UTS}$—249.9 MPa δ—75.2%	Friction coefficient—0.731 Wear loss—20.2 mg Surface roughness—0.94 µm	[58]
Zn-0.8Mn-0.4Ag	Hot Extrusion (2 µm)	$E_{corr}—$−1.19 V $I_{corr}—$11.2 µA/cm^2^ DR—0.160 mm/y	$\sigma_{TYS}—$156 MPa $\sigma_{UTS}$—251 MPa δ—63%	Nr	[113]
Zn-0.8Mn-0.4Cu	Hot Extrusion (1.1 µm)	$E_{corr}—$−1.18 V $I_{corr}—$8.91 µA/cm^2^ DR—0.133 mm/y	$\sigma_{TYS}—$191 MPa $\sigma_{UTS}$—308 MPa δ—39%	Nr	[113]
Zn-0.8Mn-0.4Ca	Hot Extrusion (2.6 µm)	$E_{corr}—$−1.16 V $I_{corr}—$10.7 µA/cm^2^ DR—0.160 mm/y	$\sigma_{TYS}—$253 MPa $\sigma_{UTS}$—343 MPa δ—8%	Nr	[113]
Zn-0.8Li-0.2Ag	Hot Rolling (2.3 µm)	$E_{corr}—$−1.21 V $I_{corr}\;—$7.67 µA/cm^2^ DR—0.11 mm/y	$\sigma_{TYS}—$196 MPa $\sigma_{UTS}$—255 MPa δ—98%	Nr	[114]
Zn-0.8Li-0.2Mg	Hot Rolling	$E_{corr}—$−1.32 V $I_{corr}—$11.3 µA/cm^2^ DR—0.17 mm/y	$\sigma_{TYS}—$254 MPa $\sigma_{UTS}$—341 MPa δ—31%	Nr	[114]
Zn-0.35Mn-0.41Cu	Hot rolling (1.1 µm)	$E_{corr}—$−1.046 V $I_{corr}—$4.1 µA/cm2 DR—0.062 mm/y (14)	$\sigma_{TYS}—$198 MPa $\sigma_{UTS}$—292 MPa δ—30%	Nr	[115]
Zn-4.3Al-3.2Cu-0.06Mg	Extrusion	$I_{corr}—$7.2 µA/cm^2^ Corrosion rate—0.374 mm/y	$\sigma_{UTS}$—201 MPa $\sigma_{TYS}—$110 MPa δ—126%	Nr	[116]
Zn-1HA	Spark plasma sintering	$I_{corr}—$21 µA/cm^2^ DR—0.327 mm/y	$\sigma_{UTS}$—158 MPa $\sigma_{TYS}—$68 MPa δ—90%	Nr	[117]
Zn-2Fe-6 v.% WC	Hot rolling	DR—0.020 mm/y $I_{corr}—$5.19 µA/cm^2^	$\sigma_{UTS}$—155.8 MPa δ—15.3% H—59.3 HV	Nr	[81]
Zn-0.5Al-0.5Mg-0.3Bi	Extrusion (30 µm)	$E_{corr}—$−1.084 V $I_{corr}—$16.45 µA/cm^2^ DR—0.203 mm/y (30)	$\sigma_{UTS}$—108 MPa δ—2.7% H—109 HV	Nr	[118]
Zn-8HA	Extrusion	DR—0.40 mm/y (14)	$\sigma_{CYS}$—113 MPa $\sigma_{UCS}$—169 MPa H—44.7 HV	Nr	[119]
Zn-3HA	Powder Metallurgy	$E_{corr}\;—$−1.070 V $I_{corr}—$5.16 µA/cm^2^ DR—0.084 mm/y	$\sigma_{CYS}$—110 MPa	Nr	[120]
Zn-16HA	Spark plasma sintering	CR—1.5 mm/y (14)	$\sigma_{CYS}$—46 MPa $\sigma_{UCS}$—65 MPa H—24 HV	Nr	[121]
Zn-5Mg	Spark plasma sintering	$E_{corr}—$−1.312 V $I_{corr}—$0.43 µA/cm^2^ DR—0.203 mm/y (50)	$\sigma_{CYS}$—183 MPa H—80.8 HV	Nr	[122]
Zn-5Mg	Powder Metallurgy	$E_{corr}—$−1.42 V DR—0.0016 mm/y (14)	$\sigma_{TYS}—$148 MPa $\sigma_{UTS}$—183 MPa δ—16% $\sigma_{CYS}$—256 MPa $\sigma_{UCS}$—209 MPa	Nr	[123]
Zn-1Mg-1TCP	Extrusion	DR—0.046 mm/y (14)	$\sigma_{TYS}—$294 MPa $\sigma_{UTS}$—330 MPa δ—11.7%	Nr	[124]
Zn-1Mg-1βTCP	Extrusion	$E_{corr}—$−1.225 V $I_{corr}—$48.9 µA/cm^2^ DR—0.732 mm/y (30)	$\sigma_{TYS}—$251 MPa $\sigma_{UTS}$—331 MPa δ—11.7%	Nr	[125]

Nr: Not reported.

## Data Availability

Not applicable.
